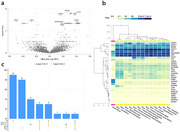# Plasma Proteomics of Cognitive Resilience to ADRD: The ARIC Study

**DOI:** 10.1002/alz70855_100152

**Published:** 2025-12-23

**Authors:** Alvin G. Thomas, Laura Raffield, Keenan A. Walker, Christy Avery, Myriam Fornage, Jan Bressler, B Gwen Windham, Ashton A. Shaffer, Maude Wagner, Anna M. Kucharska‐Newton, Alden L. Gross, Ron C. Hoogeveen, Danyu Lin, Rebecca F. Gottesman, Priya Palta, Kari E. North

**Affiliations:** ^1^ Washington University in St. Louis, St. Louis, MO, USA; ^2^ University of North Carolina at Chapel Hill, Chapel Hill, NC, USA; ^3^ National Institute on Aging, National Institutes of Health, Baltimore, MD, USA; ^4^ The Brown Foundation Institute of Molecular Medicine, McGovern Medical School, The University of Texas Health Science Center at Houston, Houston, TX, USA; ^5^ The University of Texas Health Science Center at Houston, Houston, TX, USA; ^6^ Memory Impairment and Neurodegenerative Dementia Center, University of Mississippi Medical Center, Jackson, MS, USA; ^7^ Rush University Medical Center, Chicago, IL, USA; ^8^ Johns Hopkins Bloomberg School of Public Health, Baltimore, MD, USA; ^9^ Baylor College of Medicine, Houston, TX, USA; ^10^ National Institute of Neurological Disorders & Stroke, Bethesda, MD, USA

## Abstract

**Background:**

Older adults may exhibit different levels of cognitive function despite expressing the Alzheimer's disease and related disorders (ADRD) biomarker levels. This observation, attributed to cognitive resilience, operates through unclear biological mechanisms.

**Method:**

To address this knowledge gap, we examined the association of circulating plasma proteins (SomaScan) with cognitive resilience among participants in the Atherosclerosis Risk in Communities (ARIC) study (*n* = 1,590, 58% female, 25% African American, mean age 76 (SD 5) years). We operationalized cognitive resilience as the residuals from the linear regression of general cognitive factor score (from 10 psychometric assessments) on age, sex, self‐reported race, and 20 brain magnetic resonance imaging (MRI) features. We estimated the association between 4,955 proteins and cognitive resilience (outcome) using linear regression adjusted for age, kidney function, the top ten principal components (for proteins measures), and a three‐way interaction between sex, race, and log_2_ relative protein abundance.

**Result:**

We identified 30 proteins associated with cognitive resilience overall and in race‐sex groups (Figure 1). Qiagen Ingenuity Pathway Analysis revealed that the identified cognitive resilience proteins were associated with 70 canonical pathways, including the neuroprotective DHCR24 signaling and THOP1 (biopeptide clearance) pathways. Annotated pathways suggest an important role for the brain immune system and glial cells in the biology of cognitive resilience.

**Conclusion:**

This study demonstrates the feasibility of studying cognitive resilience in a traditional cardiovascular cohort study. Further insights may be obtained through genomic and proteomic meta‐analyses leveraging consortium data.